# Reconfigurable multi-component micromachines driven by optoelectronic tweezers

**DOI:** 10.1038/s41467-021-25582-8

**Published:** 2021-09-09

**Authors:** Shuailong Zhang, Mohamed Elsayed, Ran Peng, Yujie Chen, Yanfeng Zhang, Jiaxi Peng, Weizhen Li, M. Dean Chamberlain, Adele Nikitina, Siyuan Yu, Xinyu Liu, Steven L. Neale, Aaron R. Wheeler

**Affiliations:** 1 Institute of Biomedical Engineering, University of Toronto, Toronto, ON Canada; 2 Department of Chemistry, University of Toronto, Toronto, ON Canada; 3 Donnelly Centre for Cellular and Biomolecular Research, University of Toronto, Toronto, ON Canada; 4 Department of Mechanical and Industrial Engineering, University of Toronto, Toronto, ON Canada; 5 State Key Laboratory of Optoelectronic Materials and Technologies, School of Electronics and Information Technology, Sun Yat-Sen University, Guangzhou, China; 6 James Watt School of Engineering, University of Glasgow, Glasgow, UK; 7 Photonics Group, Merchant Venturers School of Engineering, University of Bristol, Bristol, UK; 8 Present Address: Beijing Advanced Innovation Center for Intelligent Robots and Systems, Beijing Institute of Technology, Beijing, China; 9 Present Address: School of Mechatronical Engineering, Beijing Institute of Technology, Beijing, China

**Keywords:** Optical tweezers, Optical manipulation and tweezers, Optofluidics

## Abstract

There is great interest in the development of micromotors which can convert energy to motion in sub-millimeter dimensions. Micromachines take the micromotor concept a step further, comprising complex systems in which multiple components work in concert to effectively realize complex mechanical tasks. Here we introduce light-driven micromotors and micromachines that rely on optoelectronic tweezers (OET). Using a circular micro-gear as a unit component, we demonstrate a range of new functionalities, including a touchless micro-feed-roller that allows the programming of precise three-dimensional particle trajectories, multi-component micro-gear trains that serve as torque- or velocity-amplifiers, and micro-rack-and-pinion systems that serve as microfluidic valves. These sophisticated systems suggest great potential for complex micromachines in the future, for application in microrobotics, micromanipulation, microfluidics, and beyond.

## Abstract (web summary)

Light-driven micromotors can convert energy to motion in sub-millimeter dimensions. Here, the authors extend this concept and introduce reconfigurable micromachines with multiple components, driven by optoelectronic tweezers, and demonstrate new functionalities.

## Introduction

Micromotors are submillimeter particles or assemblies of particles that can convert energy into motion. There are many examples of micromotors in the literature, which can be classified on the basis of the type of energy that drives them, including magnetic^1–5^, acoustic^6,7^, electric^8,9^, and optical energies^10–16^. There is less consensus on the definition of the term “micromachine.” A working definition used here is a submillimeter system employing multiple components whose motions are coupled together to drive useful mechanical work. Although the term is used frequently in the literature, there are very few reports that satisfy this definition.

Micromotors (and potentially, micromachines) that are driven by light are particularly appealing to consider, given the maturity and diversity of beam-modulation components that can be used^17–20^, and light-driven micromotors can be subclassified into three categories based on their operating principles. The first category, opto-mechanical micromotors, relies on the interaction between light and photosensitive, mechanically responsive materials such as liquid-crystalline elastomers^21–26^. While this form of micromotor is an interesting and growing research topic, apart from demonstrations of simple functions, there are substantial material limitations that have precluded the use of these systems for many practical applications. The second category, opto-chemical micromotors, relies on photochemical reactions such as photochromic, photothermal, and photocatalytic effects to generate propulsion forces^27–31^. Opto-chemical micromotors have been demonstrated for interesting applications, but require highly specific operating conditions, including motors formed from photoactive materials and an environment that contains photochemically active reagents. The third category, optical micromotors, relies on direct manipulation of (otherwise unremarkable) microscale objects by illumination with light—the most prevalent example being optical tweezers (OTs)^17–20,32–37^. OT leverages the forces that arise from focusing light into steep intensity gradients, which permits fine and noninvasive control and actuation of microparticles suspended in air or liquid^19,20^. To date, there have been many successful demonstrations of OT-actuated micromotors that can carry out complex mechanical operations, including microfluidic pumping^38,39^, directed tissue growth^10^, and precise cell/particle translation^12,40^.

Although optical micromotors based on OT have been used for many impressive demonstrations and have many unique advantages (such as nanometer-level spatial precision^12^), OT is not a panacea for all applications. For example, OT generates forces on the order of picoNewtons (10^−12^ N), which is useful for manipulating small particles (with sizes <30 μm), but not large ones. This influences the design, structure, materials and tools used to form/fabricate the micromotors, and sets certain limits on their applications, particularly for those involving larger objects. In addition, while OT can be used to manipulate multiple micromotors in parallel, this functionality requires specialized and expensive beam-modulation hardware and control software^12,19,20,41^. This capacity was particularly important for the work described here, in which we sought to develop complex micromachines, which requires facile and robust methods for controlling many components in parallel, causing them to interact with each other.

Here, we introduce optical micromotors that are operated and controlled by optoelectronic tweezers (OET)^42–45^. OET is an optical micromanipulation technology that relies on photoconductive substrates that are typically insulating, but become conductive upon illuminating with light. By projecting illuminated and dark regions onto the photoconductive substrate, light-activated virtual electrodes can be formed in OET, which induce nonuniform electric fields producing DEP forces^42–52^. OET is capable of generation of forces on the order of nanoNetwons (10^−9^ N)^53^, which permits the manipulation of objects with sizes >100 μm^54,55^. In addition, it is particularly straightforward to use OET for parallel manipulation^41–45^, simply by projecting movies of moving shapes into a microscope. Recently, OET was used to control discrete microrobots capable of translating secondary payloads^56^. We are enthusiastic about this previous report, but note that each microrobot in the previous study achieved its functionality independently.

In this work, we sought to move beyond the single micromotor (or microrobot) to form complex, multicomponent, reconfigurable micromachines. Specifically, we describe a unit component—a light-driven micromotor that can be precisely rotated and translated—and characterize its behavior in diverse environments. We then describe how the motions of multiple micromotors can be coupled together, effectively forming light-driven, multicomponent micromachines, that can serve as 3D particle manipulators, torque and speed multipliers, and microfluidic valves. These examples are quite unique relative to what has been demonstrated previously, with potential utility for applications in hydrodynamics, microrobotics, particle/cell manipulation, and beyond.

## Results and discussion

### OET control of micromotors

**Fig. 1:**
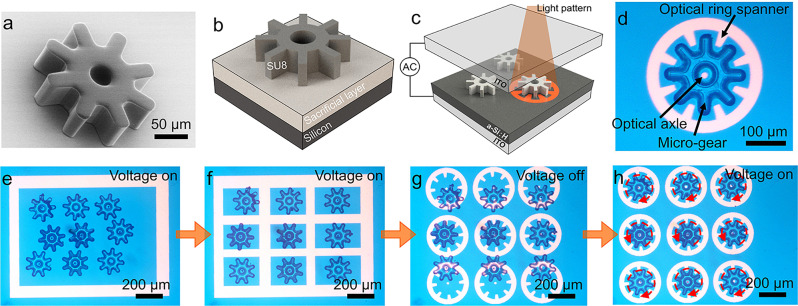
OET control of micromotors. **a** SEM image of a 200-μm-diameter micro-gear. **b** 3D schematic structure of a micro-gear prior to release. **c** Schematic depicting the principle of manipulating a micro-gear in an OET device. **d** Microscope image of a micro-gear being manipulated in an OET device. **e**–**h** Frames from Supplementary Movie 3 illustrating the step-by-step process (represented by orange arrows) of manipulating nine micro-gears such that they can be simultaneously rotated. The red-dashed arrows in (**h**) indicate that the micro-gears are rotating clockwise at 60°/s in the upper row, counterclockwise at 60°/s in the mid row, and clockwise at 120°/s in the bottom row. This parallel manipulation process requires ~30 s.

The unit component of the light-driven micromachines described here is a circular micromotor (or micro-gear) that features eight teeth arranged symmetrically around a disc with a hole in the center. Figure 1a is a representative scanning electron microscope (SEM) image of a micro-gear, which has a diameter of 200 μm and a thickness of 60 μm (Supplementary Note 1 and Supplementary Fig. 1). Figure 1b illustrates how micro-gears are formed by photolithography, followed by release from a sacrificial layer. As illustrated in Fig. 1c, after formation, micro-gears are transferred to a microscopy-based OET system in which light from a digital micromirror device (DMD) pattern illuminator is focused onto an OET device bearing a photoconductive layer [in this case, hydrogenated amorphous silicon (a-Si:H)] to exert the actuation force. Conditions were chosen such that DEP forces are negative, which serves to push the micro-gears into regions that are not illuminated. There are many examples in the OET literature^43,49,50,57^ that use simple hollow light patterns (e.g., a doughnut) to manipulate the positions of particles in this manner. Here, we adapted an approach described previously^56^ for the manipulation of microrobots, in which the light patterns are used as opto-mechanical components. For example, as shown in Fig. 1d, a standard set of components used here is an optical axle and an optical ring spanner with teeth designed to interface with those of the micro-gear. By rotating (Supplementary Movie 1) or translating (Supplementary Movie 2) the optical components, the micro-gears can be made to rotate or translate, as well (described in detail in Supplementary Note 2). Various combinations of rotation and translation enable manipulation of many micro-gears in parallel (Supplementary Movie 3 and Fig. 1e–h), a key property that is leveraged in the micromachines described below.

**Fig. 2:**
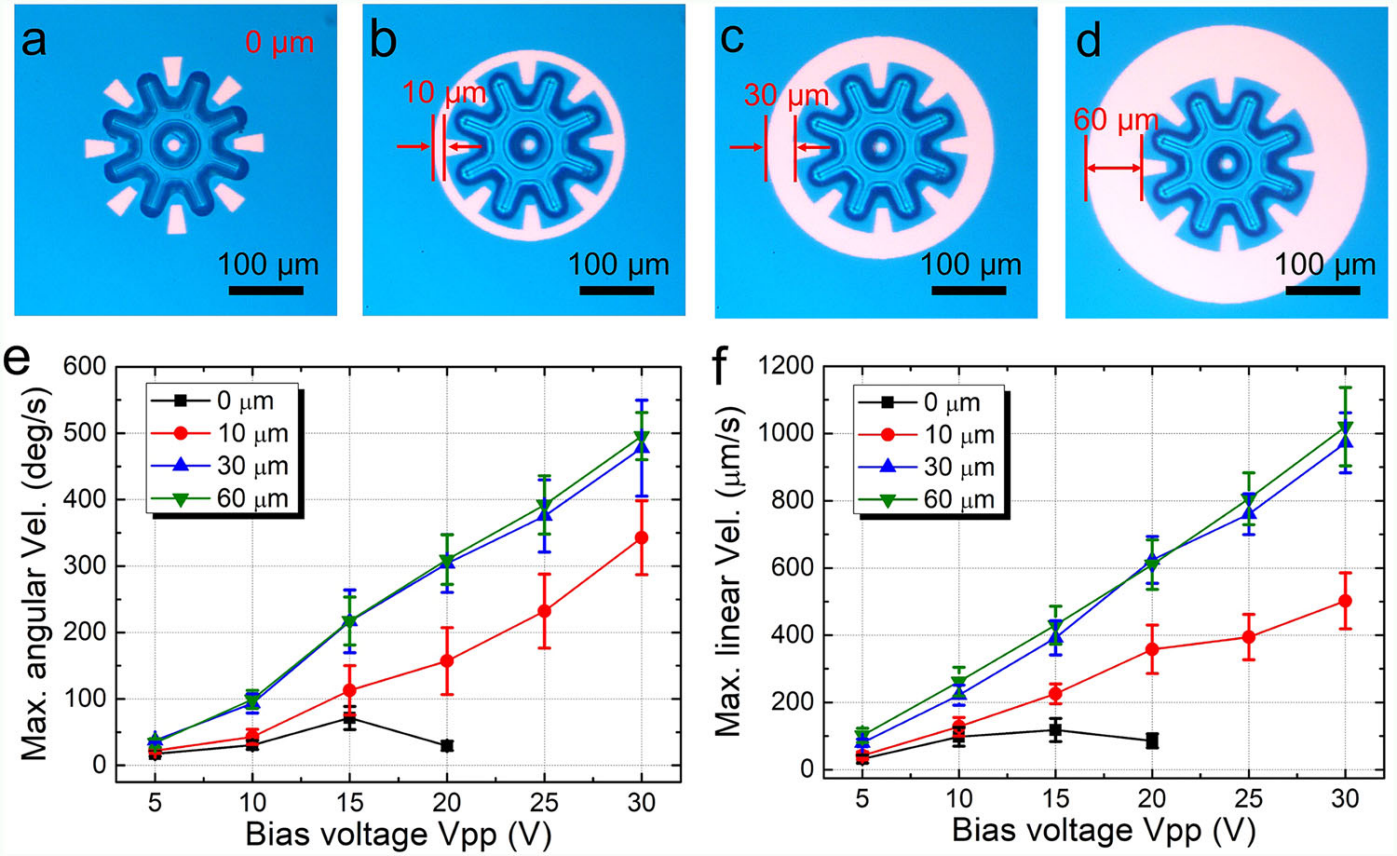
Effect of optical ring-spanner geometry on micromotors. Microscope images of micro-gears controlled by optical ring spanners with ring thicknesses of **a** 0 μm, **b** 10 μm, **c** 30 μm, and **d** 60 μm. Plots of maximum **e** angular velocity of rotating micro-gears and **f** linear velocity of translating micro-gears as a function of OET bias voltage for optical ring spanners with ring thicknesses of 0 μm (black squares), 10 μm (red circles), 30 μm (blue triangles), and 60 μm (green inverted triangles). The frequency of the OET bias was set to 10 kHz. Error bars represent ±1 SD from five measurements for each condition.

Prior to exploration of the capacity to use multiple micro-gears to form micromachines, we paused to evaluate the effect of ring-spanner geometry on micro-gear behavior. Specifically, ring spanners with ring thicknesses of 0, 10, 30, and 60 μm (Fig. 2a–d) were applied to manipulate micro-gears—when manipulated at low velocities, the micro-gears behaved as programmed. At high velocities, however, the micro-gears can no longer follow the light pattern and the operations failed. We call the condition that divides these phenomena the maximum velocity, which is plotted for rotation and translation in Fig. 2e, f. As shown, thick rings allow for much higher velocities, while micro-gears driven by thin rings fail more readily at low velocities. This result is accompanied by the observation that the failure modes differ for optical ring spanners with thin vs. thick rings. Micro-gears driven by spanners with thin rings were prone to a flipping failure mode (Supplementary Movie 4, clips 1 and 2), in which the micro-gear flipped up into the Z-dimension. In contrast, micro-gears driven by spanners with thick rings failed only by a stripping mode (Supplementary Movie 4, clip 3), akin to the mechanical stripping that is observed for a mechanical wrench that is rotated too rapidly. The flipping phenomenon, in particular, is unique, as it (unlike any other result discussed herein) is also occasionally observed in cases in which the bias voltage is applied, but the light pattern is not projected into the system (Supplementary Fig. 2). We describe this and other observations in more detail in Supplementary Note 3; more study is merited to probe these interesting phenomena.

### Micromotor impellers

**Fig. 3:**
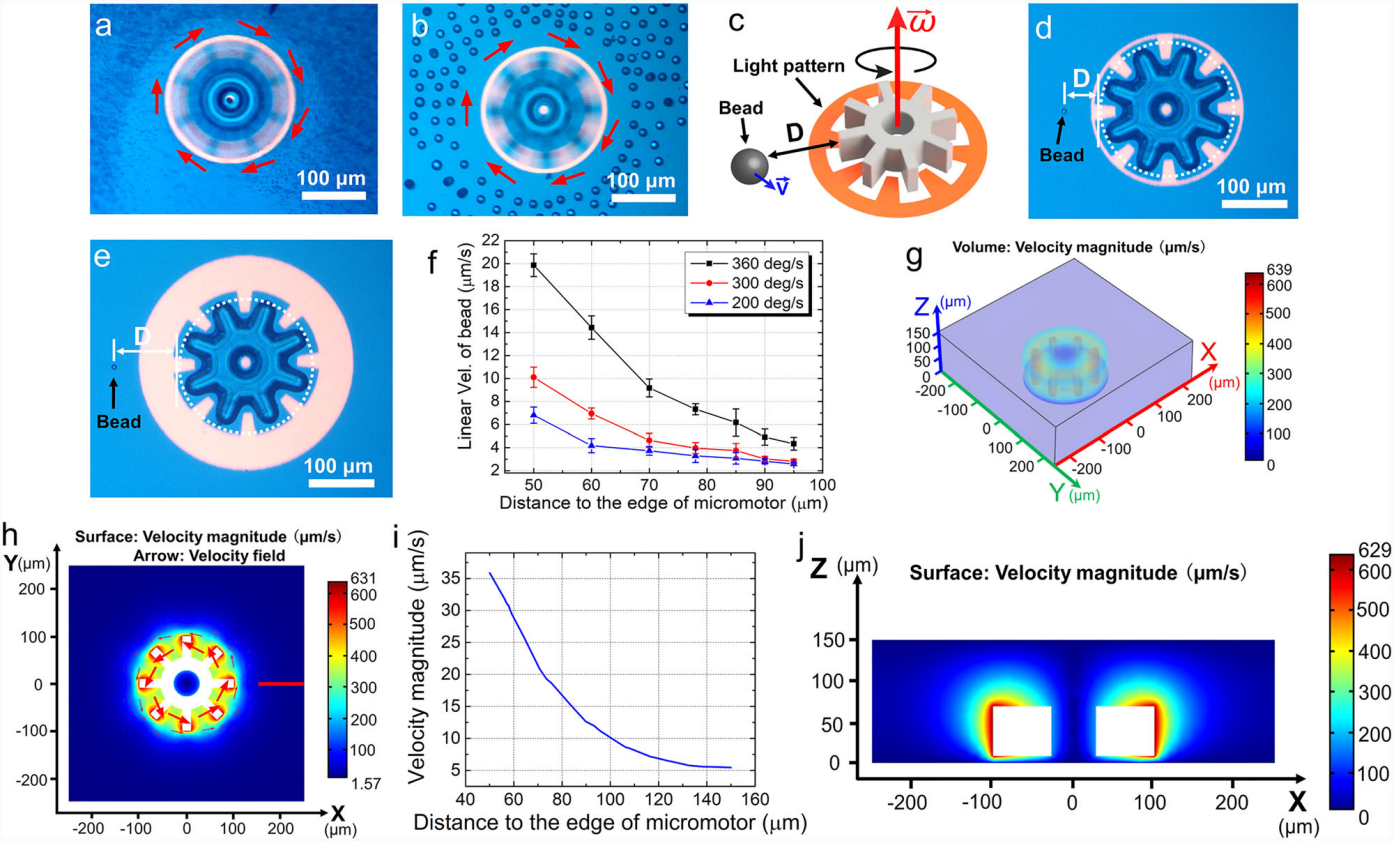
Micromotor impellers. Frames from Supplementary Movie 5 of a micro-gear rotating at 360°/s in a suspension of **a** 1-μm-diameter and **b** 15-μm-diameter polystyrene microbeads. Red arrows indicate the direction of rotation of the micro-gear and revolution of the particles. **c** Schematic of a rotating micro-gear (with angular velocity *ω*) and its influence on a nearby particle (with linear velocity *v*), and distance *D* between the two. Microscope images of a 7-μm-diameter polystyrene microbead and a micro-gear with **d** *D* = 50 μm and **e** *D* = 95 μm. **f** Linear velocities of 7-μm-diameter beads revolving around rotating micro-gears as a function of *D* for micro-gears rotated at 200°/s (blue triangles), 300°/s (red circles), and 360°/s (black squares). Error bars represent ±1 SD from three measurements for each condition. 3D (**g**), 2D (**h**), 1D (**i**), and 2D (**j**) plots of numerical simulation of the velocity of flow (in μm/s) around a micro-gear rotating at 360°/s after 2 s of rotation. Velocities in (**g**, **h**, and **j**) are shown as a heat map from 0 μm/s (blue) to over 600 μm/s (red), and arrows in (**h**) are velocity vectors. **h** is an XY slice from (**g**) at *Z* = 11 μm, **i** is an X slice of (**h**) [denoted by the red line in (**h**)], and **j** is an XZ slice from (**g**) at *Y* = 0 μm (the white boxes denote the position of the micro-gear).

With a unit component and its behavioral boundary conditions in hand, we turned to exploring the use of micro-gears as micromotors and micromachines. A common motif in the optical micromotor literature is the use of a spinning particle as an impeller, which generates hydrodynamic forces that influence the behavior of nearby particles^10,12,34,39,40^. The system described here behaves similarly—as shown in Supplementary Movie 5 and in Fig. 3a, b, a rotating micro-gear causes polystyrene microparticles in the vicinity to revolve around it. We decided to explore this phenomenon, and as illustrated in Fig. 3c, two variables were probed in these experiments—the distance *D* from the particle to the micro-gear, and the angular velocity of the micro-gear. The former variable was controlled by varying the ring thickness in the optical ring spanner. As shown in Fig. 3d, e, the negative DEP force in this system establishes a minimum value for *D*, providing convenient means to control this parameter. The latter variable was controlled by rotating optical ring spanners and micro-gears at different angular velocities, as illustrated in Supplementary Movie 6 (clips 1 and 2). Importantly, control experiments were implemented to confirm that beads do not move when exposed to a rotating optical ring spanner only, with no micro-gear (Supplementary Movie 6, clip 3). Figure 3f shows the linear velocities observed for 7-μm-diameter polystyrene microbeads as they revolve around rotating micro-gears. As shown, the bead velocities have a strong dependence on both variables—decreased distance between particle and micro-gear, and increased angular velocity of micro-gear—both cause the beads to predictably revolve at higher linear velocities.

To better contextualize the experimental results, 3D numerical simulations of fluid flow around a rotating micro-gear were carried out in COMSOL Multiphysics. In this model, a simulated micro-gear was rotated continuously for 2 s to generate a stable hydrodynamic flow (see the detailed description of the model in Supplementary Note 4 and Supplementary Fig. 3). An animation of a 3D flow velocity distribution in time for a micro-gear rotated at 360°/s can be seen in Supplementary Movie 7, and a still image from the animation at *t* = 2 s is shown in Fig. 3g. Two-dimensional slices can be generated from the simulation and visualized in Supplementary Movie 8 (XY slice for *Z* = 11 μm). A still image from this animation at *t* = 2 s is shown in Fig. 3h. A one-dimensional (X) slice can also be generated, as shown in Fig. 3i. The simulation indicates that there exists a localized field of vortex flow that drops off rapidly with increased distance from the micro-gear, which matches the experimental observations. In fact, the simulation (Fig. 3i) has a close agreement with the experiments (Fig. 3f)—for example, a 7-μm-diameter polystyrene bead located 50 μm from a micro-gear rotating at 360°/s has a linear velocity of around 20 μm/s, while the simulated fluid velocity under the same conditions is ~35 μm/s. (Note that the bead’s velocity should be reduced relative to that of the fluid, because of viscous drag.) Finally, flow velocity distribution in the vertical plane can be observed in Fig. 3j [XZ slice for *Y* = 0 μm of Fig. 3g], and the torque in this simulation at *t* = 2 s was 1.6 × 10^−13^ N⋅m.

### Micromachine 1: the touchless micro-feed-roller

**Fig. 4:**
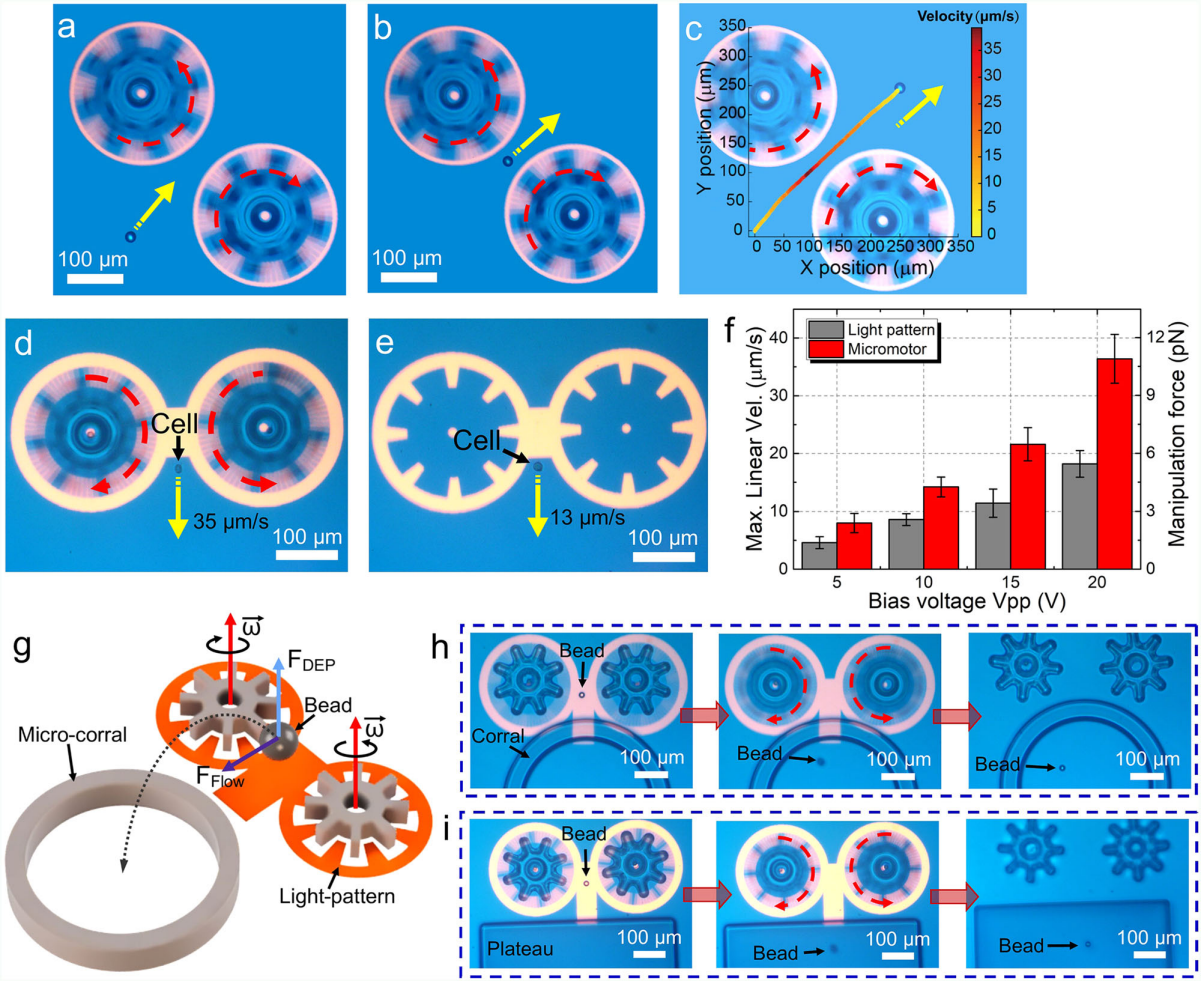
Micromachine 1: the touchless micro-feed-roller. Frames from Supplementary Movie 9 at time **a** *t* = 0 s, **b** 12 s, and **c** 27 s, depicting the position of a 20-μm-diameter polystyrene microbead propelled (with the direction indicated by yellow arrows) by a touchless micro-feed-roller. A plot of Cartesian coordinates of the bead from *t* = 0 to 27 s is superimposed on the image in (**c**), with bead velocity indicated in a heat map (yellow = stationary, red = 40 μm/s). Frames from Supplementary Movie 10 depicting the translation of a B-16 cell translated at **d** 35 μm/s by an OET-bridged touchless micro-feed-roller and **e** 13 μm/s by the light pattern alone. **f** Plot of maximum translation velocity (left axis) and corresponding force (right axis) as a function of bias voltage for the manipulation of B-16 cells by OET-bridged touchless micro-feed-roller (red) and light pattern (OET) alone (gray). Error bars represent ±1 SD from five measurements for each condition. **g** Schematic of an OET-bridged touchless micro-feed-roller used to cause a microbead to hop into a circular micro-corral. Frames from Supplementary Movie 11 illustrating the use of an OET-bridged touchless micro-feed-roller to cause a 15-μm-diameter microbead to (**h**) hop into a circular micro-corral and (**i**) hop onto a micro-plateau. Dashed red lines with arrowheads indicate the direction of rotation of micromotors in relevant panels.

The vast majority of micromotor reports in the literature^10,11,14,34–38^ (as well as the aforementioned OET microrobot^56^) describe the activity of a single micromotor (or what we reference as a unit component, as explored in Figs. 1–3). But we hypothesized that multicomponent micromachines relying on the coordinated behavior of more than one micromotor in tandem could provide additional functionalities, and we first tested the behavior of two micro-gears close together that are rotated in opposite directions. We call this a micro-feed-roller, by analogy to the pairs of cylinders that rotate to move the paper through a desktop printer. Unlike a conventional feed-roller, this system is touchless because the objects that are manipulated, such as the polystyrene beads in Supplementary Movie 9 and Fig. 4a, b, do not contact the rollers. In a hydrodynamic system such as this one, the physical extent of the control flow field is governed by the distance between the flow-actuating elements and the target objects. As shown in Fig. 4c, a particle translated by the touchless micro-feed-roller first accelerates and then decelerates, and the velocity of the particle is highest when it is in closest proximity to the micro-gears (in the midpoint of its path). This behavior is similar to what was reported previously for an OT-driven micromachine, which was found to be able to program particle position with nanometer spatial precision^12^.

The touchless micro-feed-roller is useful for moving particles across short distances; it can be modified to manipulate particles across large distances by projecting an OET pattern between the two rotating micro-gears and translating the entire system. We call this structure, shown in Supplementary Movie 10, clip 1 and Fig. 4d, an OET-bridged touchless micro-feed-roller, which combines OET manipulation with hydrodynamics. This system is particularly useful for manipulating particles that show weak responses to OET/DEP forces, such as mammalian cells. Specifically, when OET/DEP alone (with no hydrodynamics) is used to translate a B-16 cell, as in Supplementary Movie 10, clip 2 and Fig. 4e, the maximum linear velocity is slow, as viscous drag readily overwhelms the weak OET/DEP forces at high velocities. But when hydrodynamics is added to the system, as in the OET-bridged touchless micro-feed-roller, cells can be manipulated more rapidly and with greater force (calculated from velocity via Stokes’ law^48,57^), as illustrated in Fig. 4f for a range of different conditions.

Most importantly, in exploring the behavior of the OET-bridged touchless micro-feed-roller, we discovered a unique property that extends the functionality beyond two dimensions. Specifically, as illustrated in Fig. 4g, by carefully tuning the *Z*-axis DEP force^58,59^ from the OET-bridge and the *XY*-axis hydrodynamic force of the micro-feed-roller, objects can be made to controllably hop in three dimensions. (Note that previous demonstrations of similar systems have been exclusively 2D^12^.) To probe this capacity, SU-8 micro-structures were formed to serve as 3D obstacles (Supplementary Fig. 4). As shown in Supplementary Movie 11, clip 1 and Supplementary Fig. 5a, b, *XY*-manipulation using a simple OET doughnut-shaped light pattern causes microbeads to remain in one plane such that they crash into the edges of micro-structures, becoming dislodged. In contrast, as shown in Supplementary Movie 11, clip 2, the *XYZ*-forces generated by the OET-bridged touchless micro-feed-roller reproducibly cause particles to hop over (Supplementary Fig. 5c), into (Fig. 4h and Supplementary Fig. 5d) or onto (Fig. 4i) various kinds of micro-structures. As described in Supplementary Note 5, the vertical displacement (in the *Z*-dimension) at the apogee of each hop was estimated to be ~135 μm. Finally, as shown in Supplementary Movie 11, clip 3, when the *Z*-axis OET/DEP force is applied without the hydrodynamic force of the micro-feed-roller (i.e., with no operation of the micromotor), the bead hops, but does so uncontrollably (and cannot be propelled over, onto, or into a micro-structure)—the hydrodynamic force is necessary to control and predict the position of the bead. We are unaware of any other micromachine that affords this type of 3D control over secondary particles, which is analogous to a “basketball shot” or a “missile launch.” This capacity should allow for great potential to construct new variations of three-dimensional microstructures for a wide range of applications.

### Micromachine 2: the micro-gear train

**Fig. 5:**
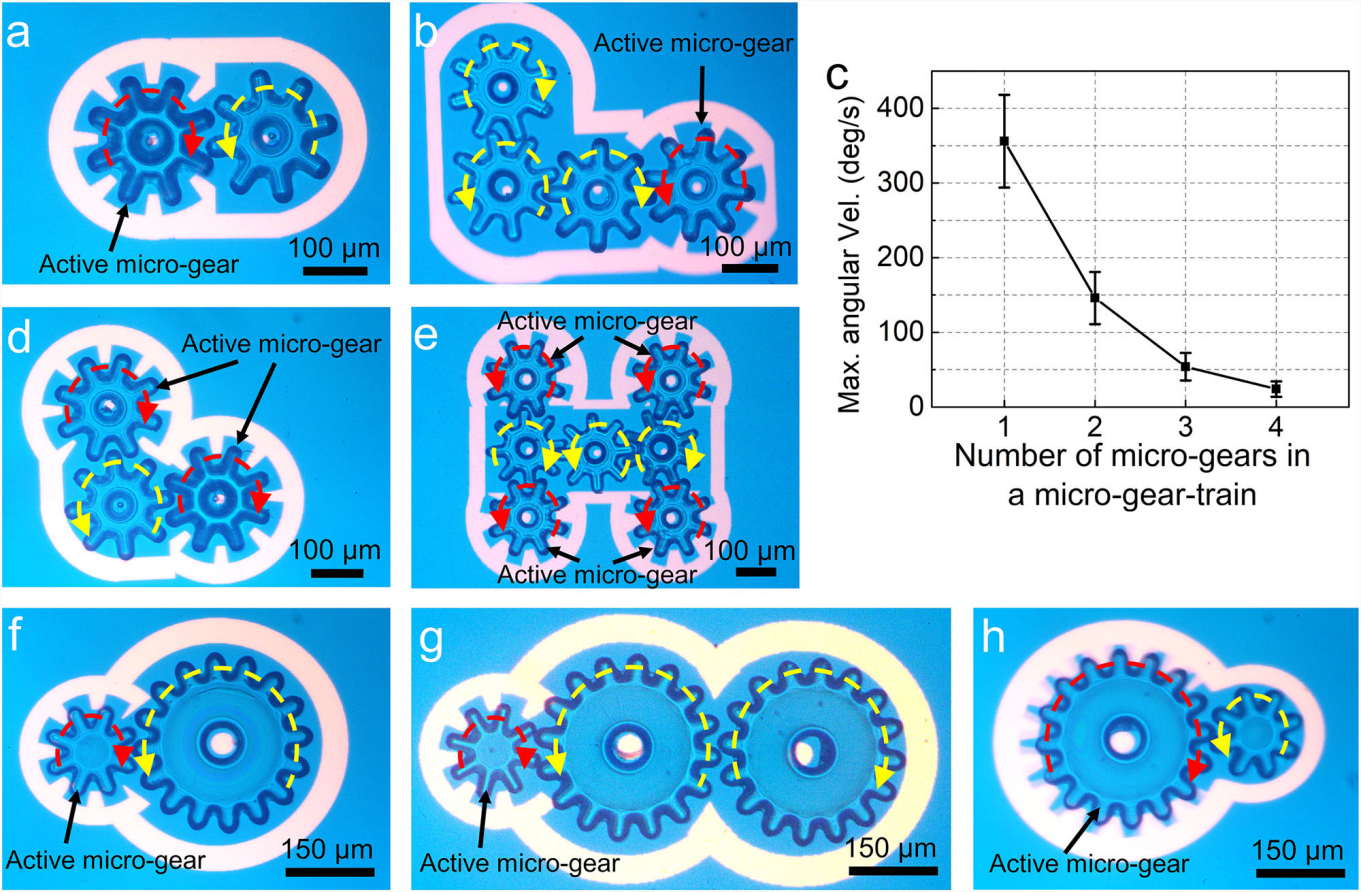
Micromachine 2: the micro-gear train. Frames from Supplementary Movie 12 illustrating micro-gear-trains driven by one active micro-gear comprising **a** two and **b** four micro-gears. **c** Plot of maximum angular velocity as a function of the number of components in a micro-gear-trains with one active micro-gear driven by a 20 *V*_pp_ OET bias voltage at 10 kHz. Error bars represent ±1 SD from five measurements for each type of micro-gear-train (assembled from different components). Frames from Supplementary Movie 13 illustrating micro-gear-trains driven by **d** two active micro-gears (three total) and **e** four active micro-gears (seven total). **f**–**h** Frames from Supplementary Movie 14 illustrating micro-gear-trains formed from small (150 μm diameter) and large micro-gears (300 μm diameter). In (**f**, **g**), the small micro-gear drives the system; in (**h**), the large micro-gear drives the system. In all images, red and yellow dashed lines with arrowheads represent the rotation directions of the active and passive micro-gears, respectively.

As a next step, we decided to investigate whether the multicomponent micromachines introduced here can provide a mechanical advantage. Specifically, we explored the possibility of driving the rotation of one or more micro-gears with optical ring spanners and then using the torque generated by this active process to drive the rotation of one or more passive micro-gears in a micro-gear train. We are aware of only a single previous report^16^ of a similar system (driven by OT), which was purely descriptive, with no details about gear size, rotation velocity, or mechanical advantage. For the current system, Supplementary Movie 12 illustrates the behavior of micro-gear trains comprising two (Fig. 5a) or four (Fig. 5b) micro-gears. A single active micro-gear generates a finite torque (e.g., we estimate 1.6 × 10^−13^ N⋅m for a micro-gear rotating at 360°/s) that can be used to drive a micro-gear train. As expected, each additional passive micro-gear increases the viscous stress and friction for the micromachine, reducing the maximum angular velocity of the system—this effect was measured and is shown in Fig. 5c. These limits can be overcome by using multiple active micro-gears to drive a gear train, as illustrated by Supplementary Movie 11 for three (Fig. 5d) and seven (Fig. 5e) micro-gears.

As described in Supplementary Note 6, the micromachines in Supplementary Movies 12 and 13 and Fig. 5a, b, d, e, which are formed from micro-gears with the same size, have a mechanical advantage MA = 1. To test the capacity to form torque multipliers with *MA* > 1 or speed multipliers with MA < 1, micro-gears with varying sizes (Supplementary Fig. 6) were formed and assembled into micro-gear trains. Supplementary Movie 14, clips 1 and 2 (Fig. 5f, g) depict micromachines with MA = 2, which amplify the input torque two-fold. Supplementary Movie 14, clip 3 (Fig. 5h) depicts a micromachine with MA = 0.5, which amplifies the input angular velocity by a factor of 2. Specifically, in this example the active gear was rotated at 100°/s, causing the passive gear to rotate at 200°/s (measured frame to frame in Supplementary Movie 14). This behavior was reproducible across multiple micro-gear trains (comprising different combinations of large and small gears), suggesting exciting possibilities for more complex light-driven micromachines in the future.

### Micromachine 3: the micro-rack-and-pinion

**Fig. 6:**
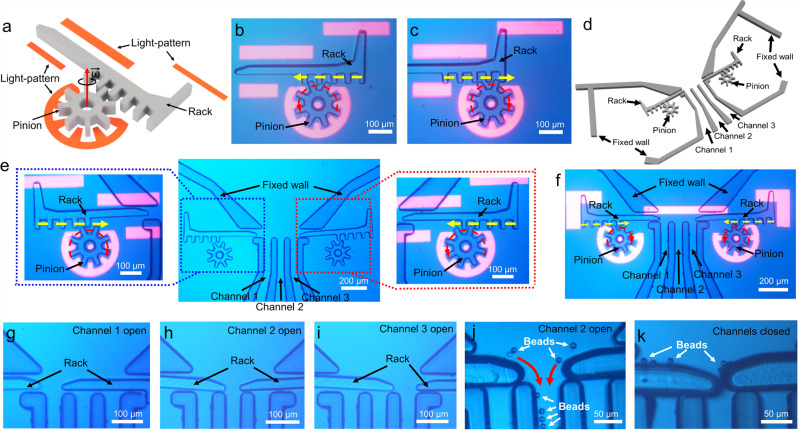
Micromachine 3: the micro-rack-and-pinion. **a** Schematic of a micro-rack-and-pinion system. **b**, **c** Frames from Supplementary Movie 15 illustrating a micro-rack-and-pinion system in operation, in which rotating the pinion counterclockwise or clockwise can make the rack translate to the left or the right, respectively (with the direction indicated by yellow dashed arrows). **d** Schematic of a permanent microchannel structure designed to interface with micro-rack-and-pinion systems. **e** Microscope images of a microfluidic valve formed from two micro-rack-and-pinion systems that can be individually controlled. **f**–**i** Frames from Supplementary Movie 15 illustrating how two rack and pinion structures can work together to choke and isolate flow in the microchannels. **j**, **k** Frames from Supplementary Movie 16 illustrating the use of micro-rack-and-pinion structures as valves to control the movement of 10-μm-diameter microbeads in microfluidic channels. In (**b**, **c**, **e**, **f**), red and yellow dashed lines with arrowheads represent the motion of the (active) pinions and the (passive) racks, respectively. In (**j**), red lines with arrowheads represent the moving direction of the microbeads.

Finally, we turned our attention to a different type of multicomponent micromachine—a micro-rack-and-pinion system (Fig. 6a) that transforms rotary into linear motion. In this system, a standard micro-gear (operated as described above) serves as the pinion, while a new component—a linear gear with commensurate dimensions (Supplementary Note 7 and Supplementary Fig. 7a, b)—serves as the rack. As illustrated in Supplementary Movie 15, clips 1 and 2, upon engaging the two components, rotating an optical ring spanner counterclockwise (Fig. 6b) or clockwise (Fig. 6c) causes the (active) pinion to rotate, which causes the (passive) rack to move linearly to the left or to the right, respectively.

We can foresee myriad applications of the micro-rack-and-pinion system, which is (to our knowledge) entirely unique in the micromotor/machine literature. As an example, a custom microfluidic structure featuring three parallel microchannels (Fig. 6d and Supplementary Fig. 7c–h) was designed and integrated into the OET chamber to evaluate the potential of using micro-rack-and-pinion systems to serve as a variable control valve. Specifically, as illustrated in Supplementary Movie 15, clips 3–6, when oriented appropriately, rotating pinions on either side of the microfluidic structure causes micro-racks to penetrate the microchannel junction (Fig. 6e, f), impeding the flow within them. By controlling the two micro-rack-and-pinion structures in tandem, the system can be configured to isolate the flow into any of the three channels in the system when driven by flow rates of up to 5 μL/min (Fig. 6g–i). As an example, the valve state can be used to control the flow of a suspension of microparticles, as illustrated in Supplementary Movie 16 (Fig. 6j, k). We note that there have been many useful types of mechanical microfluidic valves reported over the years^60–65^, but the system illustrated in Fig. 6 is the first example that we are aware of that is realized using multicomponent micromachines on-chip. Most importantly, the valves shown here are just an example. We propose that the general principles illustrated in Figs. 3–6, which include 3D particle control, torque/speed multiplication, and mechanical conversion of rotary/linear motion, may eventually be useful for applications that have not yet been imagined in hydrodynamics, microrobotics, microfluidics, and beyond.

In sum, we have introduced a new member of the micromotor family—gear-shaped components that can be rotated and translated by light-driven dielectrophoresis. Like micromotors described previously, the micro-gear components described here can be made to serve as impellers to generate localized hydrodynamic forces to control the motion of micro-objects in their vicinity. In an advance relative to the state of the art, micro-gears can be made to work together as micromachines, allowing for 3D particle control, mechanical advantage, and microfluidic valving behaviors to be realized. We propose that this represent an important step forward for the microsystems community, opening the door to ever more complex and useful micromachines in the future.

## Methods

### OET system and characterization

The OET setup comprises a DMD-based pattern illuminator (Mightex Polygon 1000G, 625 nm 1100 mW LED source) interfaced with an upright optical microscope (Leica DM 2000 microscope integrated with Märzhäuser Scan Plus 100 × 100 motorized stage), using a ×10 or ×5 objective. The optical power densities of light patterns projected through the microscope were measured using a Thorlabs PM16-130 power meter to be 0.44 and 0.12 W/cm^2^ for the ×10 objective and ×5 objective, respectively (and no optical-induced heating was observed for these conditions). Finally, the resolution of optical patterns (defined as the lateral distance between two distinguishable optical points generated by adjacent DMD pixels) formed in this system are 0.8 and 1.5 μm for the ×10 objective and ×5 objective, respectively. OET devices were similar to those reported previously^49,51,56^, consisting of a 20 μL fluidic chamber sandwiched between two glass plates separated by a 150 μm spacer. The plates are coated with a thin layer of indium tin oxide (ITO), and the bottom plate is coated with an additional layer of a-Si:H (see Fig. 1d). AC potentials (5–30 *V*_pp_, 10–30 kHz, sine waves) used to drive the OET device were supplied by a function generator (Agilent 33220A) and an amplifier (Thurlby Thandor Instrument WA31). At these frequencies, the sign of the real part of the Clasius–Mossotti factor is negative for the particles (photoresist micromotors, polystyrene beads, and biological cells) and liquid media (aqueous solutions with conductivity <30 mS/m) used here. When not specified otherwise, the OET bias was 30 *V*_pp_ at 10 kHz. In this frequency regime, DEP is the dominant force relative to AC electroosmosis^66^, and was used here because of empirical observation of high-fidelity micromotor movement in response to light pattern positions. SEM images were collected using an environmental SEM (QUANTA FEG 250 ESEM), with a pressure of 130 Pa and an electron beam with 10 keV energy and 3 nm spot size. 3D profiles were measured by an optical profilometer (Bruker Contour GT-K).

### Microfabrication

Micro-gears (including circular and linear/rack-style gears) were formed from SU-8 2050 (MicroChem, USA) at the University of Toronto’s Centre for Research and Applications in Fluidic Technologies (CRAFT) cleanroom facility using methods similar to those reported previously^56^. Briefly, 1 mL Omnicoat (MicroChem, USA) was spin-coated on top of a 4 in. silicon wafer (2000 r.p.m. for 30 s), followed by soft baking at 200 °C for 1 min. After cooling the silicon wafer to room temperature, 4 mL SU-8 2050 was spin-coated at 2500 r.p.m. (for ~60-μm-thick micro-gears) or 3500 r.p.m. (for ~30-μm-thick micro-gears) for 30 s atop the silicon substrate, followed by soft baking at 65 °C for 3 min and 95 °C for 8 min. Next, a mask aligner (OAI model 30) was used to illuminate the substrates (exposure energy: 9 mJ/cm^2^ for 20 s) through a photomask to selectively photo-crosslink the SU-8. After postexposure baking (65 °C for 2 min and 95 °C for 7 min) and developing in SU-8 developer (8 min), the substrates were air-dried with pressurized nitrogen. The substrates were then immersed in Remover PG (Microchem, USA) to dissolve the Omnicoat (3 min under gentle agitation) and to lift the photo-cured SU-8 micro-gears into suspension. The suspension was collected into a 15 mL tube and centrifuged at 14,500 × *g* for 30 s, the supernatant was removed, and the micro-gears were resuspended in bead medium [deionized (DI) water containing 0.05% volume ratio of Tween-20, P9416, Sigma] or sucrose medium [DI water, 9 wt% sucrose, 0.3 wt% d-glucose, 1.25% (v/v) phosphate-buffered saline (PBS)]. The centrifuge/resuspension process was repeated three times, after which the suspensions were stored at room temperature until use. Immediately prior to use, each micro-gear suspension was gently vortexed and an aliquot (15 μL) was loaded into the chamber of an OET device, followed by loading a second aliquot (5 μL) of bead or cell suspension.

### Sample preparation

Suspensions of polystyrene microparticles of different diameters (1, 3, 7, and 15 μm, Polysciences) were formed at 0.1–1 × 10^7^ particles/mL in bead medium (with a conductivity of 5.0 mS/m) prior to loading into OET devices. B-16 cells were cultured in DMEM (Life Technologies) with 10% (v/v) fetal bovine serum (Gibco), and 1% (v/v) penicillin and streptomycin (Invitrogen) in cell culture flasks in a humidified incubator with 5% (v/v) CO_2_/air at 37 °C. Prior to experiments, cells were washed twice in PBS (Life Technologies), passaged in 0.25% Trypsin-EDTA (Life Technologies), and centrifuged and resuspended at 0.1–1 × 10^6^ cells/mL in sucrose medium (with a conductivity of 22.1 mS/m). Finally, suspensions were filtered through a 40-μm cell strainer (Falcon) prior to loading into OET devices.

## Supplementary information

Supplementary Information

Description of Additional Supplementary Files

Supplementary Movie 1

Supplementary Movie 2

Supplementary Movie 3

Supplementary Movie 4

Supplementary Movie 5

Supplementary Movie 6

Supplementary Movie 7

Supplementary Movie 8

Supplementary Movie 9

Supplementary Movie 10

Supplementary Movie 11

Supplementary Movie 12

Supplementary Movie 13

Supplementary Movie 14

Supplementary Movie 15

Supplementary Movie 16

## Data Availability

The data generated in this study are provided in the Supplementary information/Source data file. Source data are provided with this paper.

## Source data

Source Data
