# Supplementary material for: Reconfigurable multi-component micromachines driven by optoelectronic tweezers
Source: Nat Commun. 2021 Sep 9;12:5349. doi: 10.1038/s41467-021-25582-8 (PMC8429428; doi:10.1038/s41467-021-25582-8)
Supplement: Supplementary file 1 — Supplementary Information [file 41467_2021_25582_MOESM1_ESM.pdf]

Supplementary Information for

Reconfigurable Multi-Component Micromachines Driven by Optoelectronic Tweezers

Shuailong Zhang,[1,2,3] Mohamed Elsayed,[1,3] Ran Peng,[4] Yujie Chen,[5] Yanfeng Zhang,[5] Jiaxi Peng,[1,2,3] Weizhen Li,[6] M. Dean Chamberlain,[1,2,3] Adele Nikitina,[1] Siyuan Yu,[5,7] Xinyu Liu,[4] Steven L. Neale,[6] and Aaron R. Wheeler[1,2,3]*

1. Institute of Biomedical Engineering, University of Toronto, Toronto, ON M5S 3E1, Canada.
2. Department of Chemistry, University of Toronto, Toronto, ON M5S 3H6, Canada.
3. Donnelly Centre for Cellular and Biomolecular Research, University of Toronto, Toronto, ON M5S 3G9, Canada.
4. Department of Mechanical and Industrial Engineering, University of Toronto, Toronto, ON M5S 3G8, Canada.
5. State Key Laboratory of Optoelectronic Materials and Technologies, School of Electronics and Information Technology, Sun Yat-Sen University, Guangzhou 510275, China.
6. James Watt School of Engineering, University of Glasgow, Glasgow, G12 8QQ, United Kingdom
7. Photonics Group, Merchant Venturers School of Engineering, University of Bristol, BS8 1UB Bristol, United Kingdom

Correspondence: Aaron R. Wheeler, Email: aaron.wheeler@utoronto.ca

Supplementary Figures

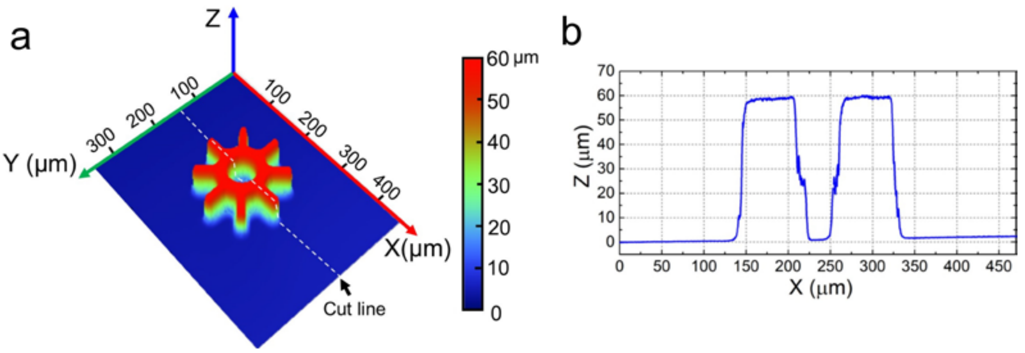

Supplementary Figure 1. Standard micro-gears. (a) 3D profile determined by optical profilometer of a micro-gear, in which the thickness is indicated in a heat map (blue = 0 μm, red = 60 μm). (b) Cross-sectional profile of the micro-gear.

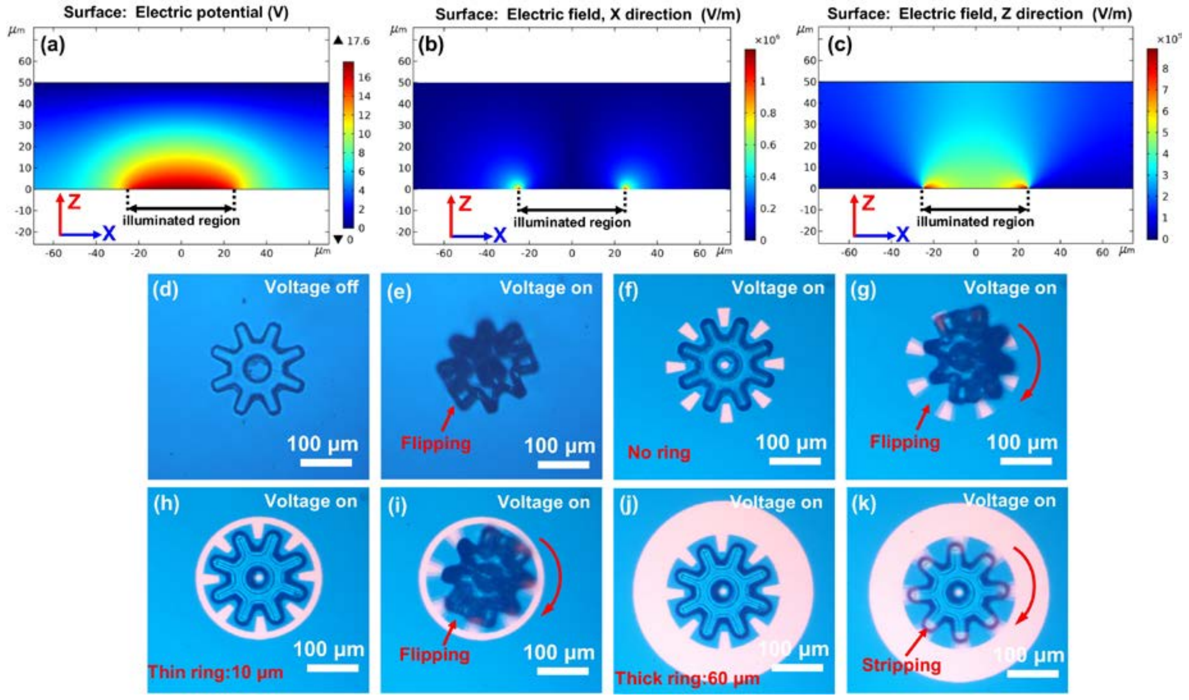

Supplementary Figure 2. Dielectrophoresis and micro-gear failure modes. Plots of (a) simulated electric potential, (b) simulated electric field in X dimension, and (c) simulated electric field in Z dimension, formed by prjecting a 50-μm-diamater circular light pattern (in the X-Y plane) on the photoconductive layer of an OET device. The simulated electric potentials and electric fields are plotted in heat maps (blue = low, red—high). Microscope images of a standard micro-gear before (d) and after (e) applying a bias voltage with no light projected. (f)-(k) Microscope images of a standard micro-gear controlled by optical ring spanners with different ring thicknesses and corresponding failure modes, with red arrows indicating the drection of rotation of the ring spanners.

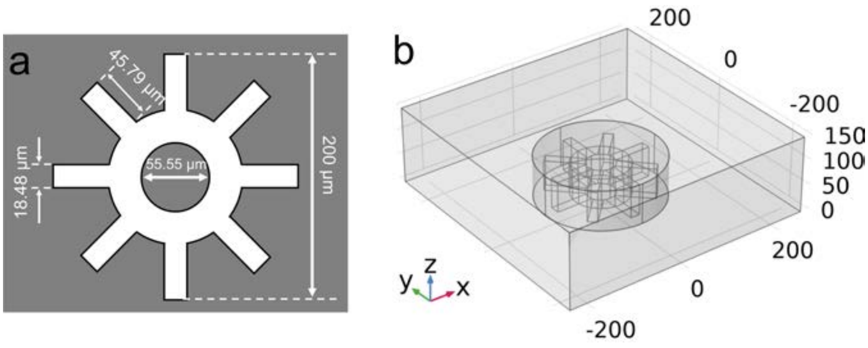

Supplementary Figure 3. Micro-gear design and simulation. (a) Design (top view) of the micro-gear. (b) Sketch of the 3D simulation model.

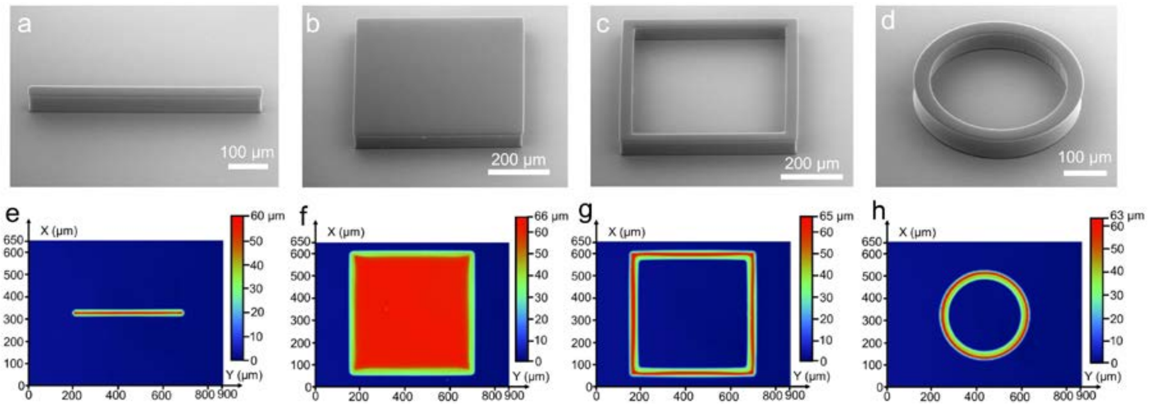

Supplementary Figure 4. Permanent micro-structures used in 3D bead manipulation experiments. SEM images of a representative (a) micro-wall, (b) micro-plateau, (c) sqaure micro-corral and (d) circular micro-corral. Meausred mechanical profiles of a representative (e) micro-wall, (f) micro-plateau, (g) square micro-coral and (h) circular micro-corral. The height is indicated in a heat map (blue = low, red = high).

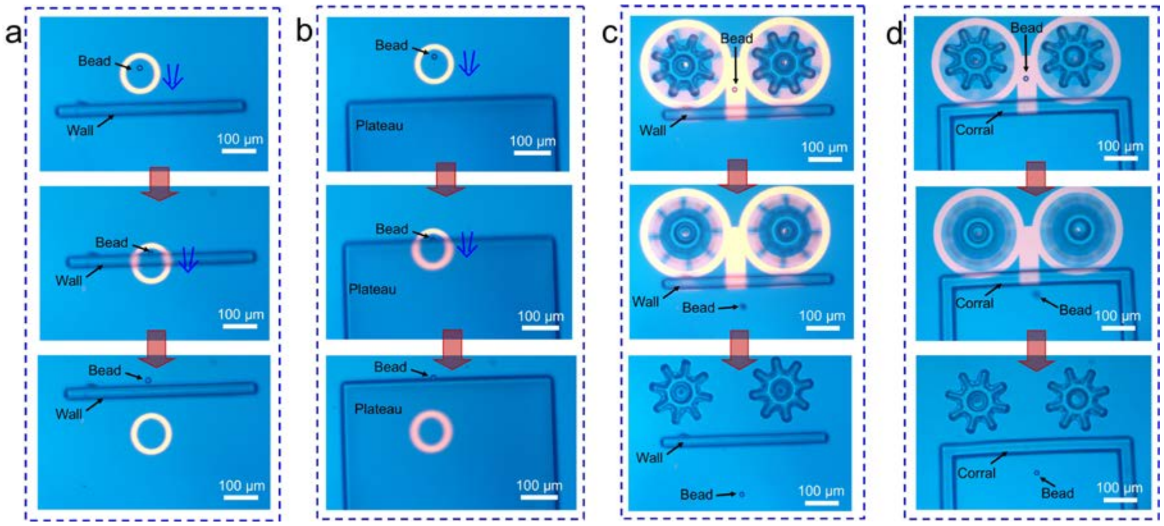

Supplementary Figure 5. Frames (top-to-bottom) from Supplementary Movie 11 illustrating manipulation of 15 μm dia. polystyrene microbeads around SU-8 micro-structures. A doughnut-shaped OET light pattern pushes particles toward (a) a micro-wall, and (b) a micro-plateau. The blue arrow indicates the direction of movement of the light pattern. An OET-bridged touchless micro-feed-roller causes particles to (c) hop and cross over a micro-wall and (d) hop into a sqaure micro-corral.

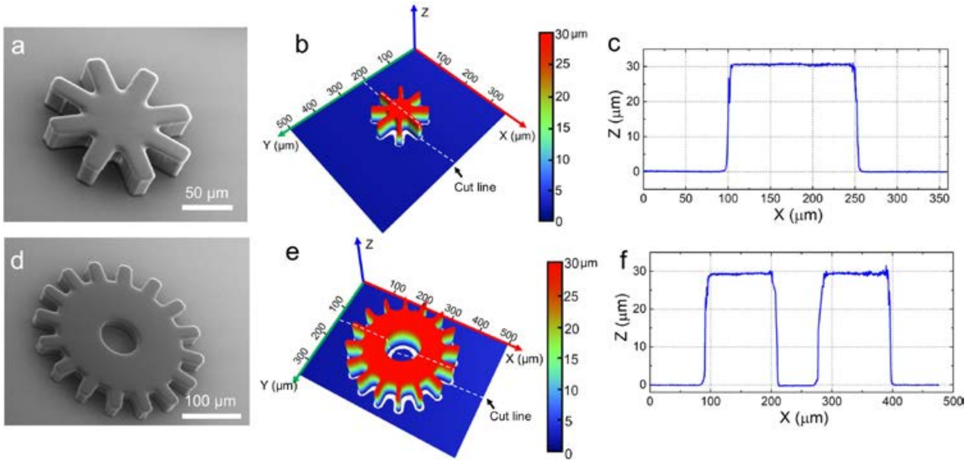

Supplementary Figure 6. Small and large micro-gears. (a) SEM image of a small micro-gear. (b) 3D profile of a small micro-gear, in which the height is indicated in a heat map (blue = 0 μm, red = 30 μm). (c) Cross-sectional profile of a small micro-gear, corresponding to the cutline in (b). (d) SEM image of a large micro-gear. (e) 3D profile of a large micro-gear, in which the height is indicated in a heat map (blue = 0 μm, red = 30 μm). (f) Cross-sectional profile of a large micro-gear, corresponding to the cutline in (e).

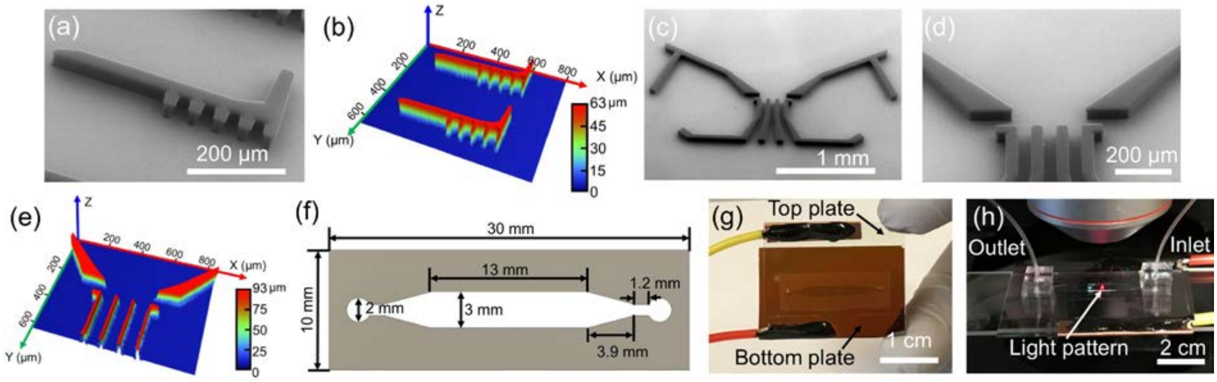

Supplementary Figure 7. Micro-rack-and-pinion components and devices. (a) SEM image of a micro-rack. (b) 3D profile of a micro-rack, in which the height is indicated in a heat map (blue = 0 μm, red = 63 μm). (c)-(d) SEM images of a butterfly-pattern SU-8 micro-structure, featuring a junction of three parallel microchannels in the center. (e) 3D profile of the center of the microchannel junction in the center of a butterfly pattern on an OET device bottom-plate, in which the height is indicated in a heat map (blue = 0 μm, red = 93 μm). (f) Design (top view) of the xurographically defined chamber used in OET devices for micro-rack-and-pinion experiments. (g) Photograph (top view) of an assembled OET device with a chamber fabricated according to the parameters in (g). (h) Photograph of an OET device for micro-rack-and-pinion experiments in operation, with the flow rate controlled via a syringe pump connected to the outlet.

Supplemntary Notes

Supplementary Note 1: Micro-gear dimensions

Supplementary Figure 1a shows the measured 3D profile of a standard micro-gear used here, in which the thickness is indicated in a heat map (blue = low, red = high). Supplementary Figure 1b shows the cross-sectional profile of the micro-gear, corresponding to the dashed-white cutline in Supplementary Figure 1a. As indicated, the width and thickness of the standard micro-gear were approximately 200 μm and 60 μm, respectively, and the overall dimensions were similar to those that were designed (Supplementary Figure 3a). These standard micro-gears were used for most experiments; large and small micro-gears were used in others, described in Suppementary Note 6, below.

Supplementary Note 2: Direct and Indirect Micro-object control

Micro-objects (including micro-gears) were directely controlled by OET by (1) applying a a sine-wave AC bias between the electrodes on the bottom and top plates of OET devices (if not otherwise specified, the magnitude and frequency was 30 V_{pp} at 10 kHz), and (2) projecting an optical image onto the bottom plate of an OET device. The order of these steps (1 & 2) was varied; objects in the chamber are not affected until both are engaged (with one exception – see the discussion in Supplementary Note 3, below). In the conditions used here the objects to be controlled experience negative DEP force[1]; thus, the images in (2) were designed to push particles into regions that were not illuminated. The images used in (2) are described in the following:

(i) The most common image was an optical ring-spanner paired with an optical axle (e.g., Figure 1d in the main text). The optical ring-spanner was a negative-relief outline of the perimeter of the standard micro-gear, comprising an illuminated hollow circle (or ring) with 8 illuminated optical teeth (isosceles trapezoid shape, shorter side: 17 μm; longer side: 25 μm; height: 40 μm) symmetrically disributed around the ring, penetrating into the hollow (dark) center. The inner diameter of the ring was 230 μm, and the ring thickness was varied (if not otherwise specified, the thickness was 30 μm). The optical axle was a 10 μm dia. illuminated circle projected in the center of the ring-spanner image. In most experiments, micro-gears were made to rotate by rotating the teeth in an optical ring-spanner relative to the device (e.g., Supplementary Movie 1); unless otherwise specified, the rotation frequency was 360 deg/s. In other experiments, micro-gears were translated by moving the device relative to a stationary image (e.g., Supplementary Movie 2, clip 1). In still other experiments, micro-gears were translated and rotated by doing both (e.g., Supplementary Movie 2, clip 2). For the majority of experiments in which manipulation was successful, micro-gear movement was found to perfectly keep up with the (relative) movement of the projected light pattern. In this case, the programmed velocity was taken as the actual velocity. When operated above the maximum velocity, the manipulation failed, as discussed in Supplementary Note 3.

(ii) Another common image used was a hollow rectangle, with inner (dark) region large enough to encompass one or more micro-gears. Micro-gears trapped within the image were further manipulated by adding sub-dividing grid-lines to the image (e.g., Supplementary Movie 3).

(iii) A touchless micro-feed-roller was formed by projecting two optical ring-spanner/axle pairs (each controlling a micro-gear) positioned 290 μm from each other (center-to-center), with optical teeth rotated in opposite directions.

(iv) An OET-bridged touchless micro-feed-roller was formed by projecting the same image from (iii), with the addition of an illuminated rectangle between the rings. For cell manipulation (e.g., Figure 4d in the main text and Supplementary Movie 10), the illuminated rectangle was 32 μm×80 μm; for microbead manipulation (e.g., Figure 4h and 4i in the main text, and Supplementary Movie 11, clip 2), the rectangle was 58 μm×180 μm. In some experiments, OET-bridged touchless micro-feed-rollers were not translated (e.g., Supplementary Movie 9); in other experiments, OET-bridged touchless micro-feed-rollers were translated by moving the device relative to the images (Supplementary Movie 10).

(v) A doughnut- (or ring-) shaped image with 68 μm inner diameter and 13 μm ring-thickness was used to manipulate polystyrene microbeads[2] (e.g., Supplementary Figure 5a-b; Supplementary Movie 11, clip 1).

(vi) Images used to control micro-gear-trains (e.g., Figure 5 in the main text and Supplementary Movie 12-14) included three sub-components: optical boundaries, optical axles, and partial optical ring-spanners. Optical boundaries are illuminated, hollow outlines (with 30 μm thickness) designed to closely circumscribe the outer perimeter of each gear-train, and optical axles are identical to what is described above. Partial optical ring spanners are similar to what is described above (with rotating optical teeth on a hollow ring used to drive the rotation of a micro-gear), but with an opening (that is not illuminated) located at the portion on the micro-gear that engages with its neighbor. Further, the optical ring thickness and diameter, as well as the number and size of optical teeth were scaled appropriately when used to control small and large gears (described in Supplementary Note 6 below).

(vii) Images used to control micro-rack-and-pinion (e.g. Figure 6 in the main text and Supplementary Movie 15) included three sub-components: optical boundaries, optical axles, and partial optical ring-spanners. Optical boundaries are illuminated straight lines (with 50 μm thickness) designed to circumscribe the outer perimeter of the pinion. Optical axles are identical to what is described above. Partial optical ring spanners are similar to what is described above (with rotating optical teeth on a hollow ring used to drive the rotation of the rack), but with an opening (that is not illuminated) located at the portion on the rack that engages with the pinion.

In addition to direct OET control, micro-objects (including polystyrene microbeads and mammalian cells) were also controlled indirectly by fluid flow driven by micro-gear rotation. Micro-objects were found to revolve around single rotating micro-gears. In such systems, linear velocities of micro-objects were calculated from the revolution time and the approximate travel-path. Micro-objects were found to travel linearly between micro-gears in touchless micro-feed-rollers, and their Cartesian coordinates and velocities were determined using Tracker (https://physlets.org/tracker/). Micro-objects were made to (indirectly) follow more complex (3D) trajectories using OET-bridged touchless micro-feed-rollers (Supplementary Note 5, below), and the rotation of secondary micro-gears was (indirectly) controlled by direct action of primary micro-gears (suppelementary note 6, below).

Supplementary Note 3: Dielectrophoretic manipulation and failure mode analysis

In an OET system, the micromanipulation force relies on dielectrophoresis (DEP), an electrokinetic phenomenon in which a non-zero force is exerted on a polarizable particle suspended in a medium when it is subjected to a non-uniform electric field. The strength of the DEP force depends on the medium's and the object's electrical

properties, on the object's shape and size, as well as on the frequency and magnitude of the electric field. The most widely used method to calculate the DEP force is the classic dipole approximation[1] in which the force acting on a spherical particle is given by the following,

$$F_{\text{DEP}} = 2\pi r^3 \varepsilon_m \operatorname{Re}[K(\omega)] \nabla E^2 \quad (1)$$

where r is the particle radius, ε_m is the permittivity of the medium, E is the amplitude of the root-mean-square (RMS) electric field, and Re[K(ω)] is the real part of Clausius-Mossotti (CM) factor. The latter is defined by the following,

$$K(\omega) = \frac{\varepsilon_p^* - \varepsilon_m^*}{\varepsilon_p^* + 2\varepsilon_m^*} \quad (2)$$

where ε_p^* and ε_{m}^* are the complex permittivities of the particle and medium, respectively. When Re[$K(\omega)$] > 0, the DEP force acting on the object is positive (such that it moves into the illuminated regions); when Re[$K(\omega)$] < 0, the DEP force acting on the object is negative (such that it moves away from the illuminated regions, as in the conditions used here). Note that equation (1) is derived from the consideration of a single molecular dipole; it is not appropriate for quantitative predictions of force acting on large structures such as the micro-motors used here. Thus, the qualitative trends predicted from equation (1) are referenced herein, but the equation was not used to generate quantitative DEP forces in this report.

COMSOL Multiphysics (COMSOL Inc., Version 5.4, Burlington, MA, accessed via license obtained through CMC Microsystems, Kingston, Canada) was used to estimate the electric field distribution in an OET device illuminated by a 50-µm-diamater circular light pattern (in the X-Y plane) using a model reported previously.[2] As shown in Supplementary Figure 2a, as predicted, fluid adjacent to the illuminated region is held at higher electric potential than the bulk solution. Likewise, there is a strong electric field gradient in the X dimension (Supplementary Figure 2b) (and also the Y dimension, not shown), which is the effect that is most often used in OET-DEP experiments. In the negative DEP regimes employed here, it is the force generated by this field gradient [approximated by equ. (1)] in the X and Y diemsnions that causes micro-gears and other particles to move to dark regions of the OET device in the plane of view. Finally, as shown in Supplementary Figure 2c, although it is often ignored in the literature, there is also a large field gradient in Z dimension,[3,4] which can influence the behaviour of particles in Z direction as well. This is fundamentally important for the work in Suppelemtnary Note 5, below.

When a rotating optical ring spanner and axle is projected into an OET device, the electric field gradient in the X-Y dimension (Supplementary Figure 2b) drives a dielectrophoretic force (eq. 1) that causes a micro-gear in the vicinity to rotate, as well, such that the micro-gear remains in the dark regions of the device. This effect is predictable at low angular velocities, but begins to fail at high velocities, such that the micro-gear does not keep up with the projection. Interestingly, one of the failure mechanisms that we observe in these cases, which we call flipping, can occasionally be observed without projecting a light pattern. Supplementary Figure 2d-e illustrates such a case - when voltage is applied (20 V_{pp} at 10 kHz) without a light projection, the mico-gear flips on its side in the Z-dimension. Similar phenomona have been reported for silver nanowires[5] and carbon nanotubes[6] in OET systems (with no light projected), which were attributed to shaped-induced anisotropic polarization. That is,

while the projection of light is typically used to generate strong electric field gradients in OET devices (Supplementary Figure 2b-c), the simple presence (in the dark) of an asymmetric object wth $\text{Re}[K(\omega)] \neq 0$ can also generate electric field gradients, in a manner similar to insulator-based DEP.[7] This may explain why micro-gear flipping is occasionally observed in the dark in the results reported here (Supplementary Figure 2d-e).

More generally, the flipping failure mode is commonly observed for micro-gears in optical ring spanners with no ring (Supplementary Figure 2f-g) or a thin ring (Supplementary Figure 2h-i), when rotated at high angular velocities. In this case, the flipping may be caused by anisotropic polarization (as in the case of no illumination), or it may be the result of the micro-gear drifting over an illuminated region with a high electric-field gradient (and thus DEP force) in the Z-dimension (Supplementary Figure 2c). Interestingly, the flipping phenomenon is not observed for micro-gears rotated in optical ring spanners with a thick ring; in these systems, micro-gears are observed to fail by a stripping phenomenon (Supplementary Figure 2j-k). We hypothesize that the different length-width scaling of DEP forces[8] in the XY and Z dimensions reduces the effect of anisotropic polarziation and Z-dimension DEP forces that might drive flipping in this case. Specifically, since the micro-gears used here are thick (60 μm height), there should be a strong XY-dimension DEP force vertically across the sidewall of the micro-gear, thus providing stronger force to confine the micro-gear in the XY-plane and prevent it from flipping. Other mechanisms that may play a role include light-induced AC electroosmosis[9] (LACE), in which ion-induced fluidic flow might exert a horizontal fluidic pressure force to the side-wall of a micro-gear to help keep it in the XY-plane. Regardless, these interesting observations suggest additional experiments and simulations for the future.

Supplementary Note 4: Numerical simulation of rotating micro-gear

The design paramegers of the standard micro-gears are shown in Supplementary Figure 3a, which was used as the basis to form a three-dimensional virtual model of a micro-gear (Supplementary Figure 3b) in a volume with length (X axis), width (Y axis), and height (Z axis) of 500 μm, 500 μm, and 150 μm, respectively. The micro-gear was positioned at the center of the model (in the X-Y plane) and 10 μm above the bottom plane (in the Z dimension), and has identical dimensions to those in Supplementary Figure 3a as well as a thickness of 60 μm. COMSOL Multiphysics was used to simulate rotation of the micro-gear, using the Rotating Machinery, Laminar Flow module. This simulation is based on the Navier-Stokes equation, which is applied to solve the flow field in the model volume. Space not occupied by the micro-gear was set to be equivalent to water (at room temperature) with dynamic viscosity and density of 0.0018 Pa•s and 1000 kg/m^3, respectively. The boundary condition at the micro-gear sidewall was set to no slip, assuming no relative fluid movement at the micro-gear sidewall. The upper and lower surface of the model were also set to no slip (i.e., no fluid movement) and the side surfaces were set to free boundaries, allowing fluid to flow in and out. In simulations of rotating micro-gears, two free tetrahedrayl mesh domains were used: a moving domain (located in the central cyllinder with radius 120 μm and height 80 μm, positioned 5 μm above the bottom plane) and a stationary domain (everywhere else), with the flow continuity condition applied to the boundary between them. The moving mesh domain had a maximum element size of 14.5 μm, a minimum element size of 2.74 μm, a maximum element growth rate of 1.13, a curvature factor of 0.5, and a resolution of narrow regions of 0.8. The stationary mesh domainhad a maximum element size of 27.4 μm, a minimum element size of 8.22 μm, a maximum element growth rate of 1.2, a curvature factor of 0.7, and a resolution of narrow regions of 0.6. With these parameters, independent and converging simultion results

were achieved in around 46 hours.

Supplementary Movie 7 shows the results allowing the model to propagate for 2 s in intervals of 0.05 s, with a starting velocity of 0 deg/s, an acceleration of 3600 deg/s^2, and a saturation velocity of 360 deg/s. Supplementary Movie 7 represents an XY slice from Animation 1 at Z = 11 μm, representing the flow distribution around the the bottom of the micro-gear. The simulated torque *T* of the micro-gear at t = 2 s was calculated to be 1.6$\times$10^{-13} N·m by integrating the fluidic viscous stress moment (X-Y plane) over the surface of the micro-gear using the following equation,[10]

$$T = \int_{Body} r \times \prod \cdot dS \quad (3)$$

in which $\prod$ is the fluidic viscous stress and r is the position factor of a point relative to the rotational axis.

Supplementary Note 5: Micro-object manipulation in three dimensions

A series of permanent micro-structures, including micro-walls, micro-plateaus, and square and circular micro-corrals, were formed from SU-8 on OET bottom plates using methods similar to what we reported previously.[11] Supplementary Figure 4a-d shows scanning electron microscope (SEM) images of representative microstructures, and Supplementary Figure 4e-h shows measured 3D profiles for the same structures, which had heights of around 60 μm.

Supplementary Movie 11 illustrates how micro-structures were used to evaluate the capacity of OET and micromotors for micromanipulation in the three dimensions. For example, a simple OET doughnut-shaped light pattern can push an object toward a micro-wall (Supplementary Figure 5a) and a micro-plateau (Supplementary Figure 5b). As shown, objects transported in this manner remain in the same plane in the Z-dimension and become trapped at the edge of the structure. In contrast, the OET-bridged touchless micro-feed-roller is capable of causing objects to hop over and into 3D structures, as shown in Supplementary Figure 5c-d. This is achieved by carefully tuning the Z-axis DEP force (driven by electric field in the Z-dimension, as in Supplementary Figure 2c) and the XY-axis hydrodynamic force. Specfically, the Z-axis DEP force can be controlled by adjusting the voltage and frequency of the OET bias, and the layout of the light pattern; the XY-axis hydrodynamic force can be controlled by adjusting the rotational velocity of the micro-feed-roller. Bead hopping height was determined as a function of apparent (out-of-focus) bead area using methods similar to those described previously.[4] Briefly, first, the microscope stage was fixed in position such that a 15 μm dia. bead at or near the bottom plate surface was in focus (Z = 0). Then, the stage was raised to Z = 60 μm to collect an image of the (out-of-focus) bead. Then, the stage was raised in 5 μm increments from Z = 60 μm to Z = 160 μm, collecting an image of the (out-of-focus) bead at each step. These data allowed for calibration of apparent (out-of-focus) bead area as a function of Z-axis position. Second, in OET-bridged touchless micro-feed-roller experiments, the area of the apparent bead-area (when most out of focus) was compared to the calibration data to estimate the Z-axis position at apogee.

Supplementary Note 6: Micro-gear-trains

Micro-gear-trains were formed from two or more micro-gears positioned such that the teeth in the gears can

engage mechanically with each other (e.g., Figure 5 in the main text). Micro-gear-trains were formed from standard micro-gears (Supplementary Figure 1), but also from small and large micro-gears (Supplementary Figure 6). Specifically, Supplementary Figure 6a-c shows an SEM image, a measured 3D profile, and a cross-sectional profile of a representative small micro-gear, and Supplementary Figure 6d-f shows analogous data for a large micro-gear. As indicated, the widths of the small and large micro-gears are approximately 150 μm and 300 μm, respectively, and the thicknesses are approximately 30 μm. (The reduced thickness was found to be important for manipulation of large micro-gears.) Note that the small micro-gear has eight teeth (like the standard micro-gear), but no through-hole (and was not used with an optical axle), whilethe large micro-gear has sixteen teeth and a through-hole (and was used with an optical axle).

Micro-gear-trains were positioned such that one or more micro-gears was active, with rotation driven by the rotation of a partial optical ring-spanner, and one or more micro-gears was passive, with rotation driven by mechanical interaction with an adjacent micro-gear (in opposite direction to that of the adjacent micro-gear). In general, for each pair of gears in a gear-train, the velocity v of the point of contact on between them is:

$$\mathrm{v} = r_{\mathrm{In}}\,\omega_{\mathrm{In}} = r_{\mathrm{Out}}\,\omega_{\mathrm{Out}} \tag{4}$$

where the driving or input micro-gear has radius r_{In} and angular velocity ω_{In}, and the output micro-gear has radius r_{Out} and angular velocity ω_{Out}. The mechanical advantage *MA* (also known as the torque ratio) is:

$$MA = \frac{\omega_{In}}{\omega_{Out}} = \frac{r_{Out}}{r_{In}} = \frac{N_{Out}}{N_{In}} = \frac{T_{Out}}{T_{In}} \tag{5}$$

where N_{Out} is the number of teeth on the output micro-gear, N_{In} is the number of teeth on the intput mico-gear, T_{Out} is the output torque, and T_{In} is the input torque.

Supplementary Note 7: Micro-rack-and-pinion systems

Micro-rack-and-pinion systems were formed from a standard micro-gear (the pinion) and a ~500 μm-long, ~63-μm thick L-shaped rack (Supplementary Figure 7a-b). Each arm of the L in the rack is ~35 μm wide, and the portion of the long arm closest to the junction features five teeth with dimensions and pitch designed to match those in a standard micro-gear. Micro-rack-and-pinion systems were positioned such that an active pinion was controlled directly by an optical ring-spanner. Rotation of the pinion caused the passive rack to move in a predictable linear pattern when surrounded by the appropriate optical boundaries (Figure 6 in the main text).

Micro-rack-and-pinion systems were designed to interface with a permanent butterfly pattern microstructure (Supplementary Figure 7c). The center of each butterfly design (Supplementary Figure 7d) features four parallel SU-8 features which together define the sidewalls of three parallel 50 μm wide microchannels. The three microchannels terminate into a large chamber, but at the junction the assembly is bisected by ~55 μm wide openings. The pattern was designed to interface with two rack-and-pinion-systems – one to the left and one to the right of the microchannel junction. When oriented properly, when the pinions rotate, the long axis of each rack penetrates the perpendicular openings at the junction, where they can choke-off the flow through the microchannels.

Each butterfly design was formed from ~93-μm thick SU-8 (Supplementary Figure 7e) on OET bottom plates using methods similar to what we reported previously.[11] OET devices bearing these structures were similar to the standard devices described in the main text, except that the top plate was modified to include two 1 mm dia. inlet- and outlet-holes, and the spacer between the top and bottom plates (formed from Adhesive Research product 90178, which is ~88 μm thick) featured a linear chamber with shape and dimension shown in Supplementary Figure 7f, formed by xurography using an automated craft cutter (Graphtec ce6000-60). Upon assembly (Supplementary Figure 7g), each device featured one butterfly SU-8 structure located in the center of the chamber, with the three parallel microchannels parallel to the long axis of the chamber. When compressed together, the two plates and the SU-8 microstructure formed a network of sealed microchannels.

Finally, devices were completed (Supplementary Figure 7h) by affixing PDMS reservoirs bearing 1 mm dia. punched holes to the top of the top plates, to interface with PVC tubing (TYGON AAQ02091, inner dia. 0.01 inch). The outlet tube was connected to a programmable syringe pump (Harvard Apparatus PHD Ultra), which was used to pump fluid through the microchannels in the butterfly structure at up to 5 μL/min. (Note: all dimensions are given as-designed; the actual structures had some variation upon fabrication and assembly.)

Supplementary References

1. R. Pethig, Dielectrophoresis: Status of the theory, technology and applications. *Biomicrofluidics* 4, 022811 (2010).
2. S. Zhang, W. Li, M. Elsayed, P. Tian, A. W. Clark, A. R. Wheeler, S. L. Neale, Size-scaling effects for microparticles and cells manipulated by optoelectronic tweezers. *Opt. Lett.* 44, 4171-4174 (2019).
3. S. Zhang, A. Nikitina, Y. Chen, Y. Zhang, L. Liu L, A. G. Flood, J. Juvert, M. D. Chamberlain, N. P. Kherani, S. L. Neale, A. R. Wheeler, Escape from an Optoelectronic Tweezer Trap: experimental results and simulations. *Opt. Exp.* 26, 5300-5309 (2018).
4. Y. Zhao, H. S. Lai, G. Zhang, G. B. Lee, W. J. Li, Rapid determination of cell mass and density using digitally controlled electric field in a microfluidic chip. *Lab Chip* 14, 4426-4434 (2014).
5. A. Jamshidi, P. J. Pauzauskie, P. J. Schuck, A. T. Ohta, P. Y. Chiou, J. Chou, P. Yang, M. C. Wu, Dynamic manipulation and separation of individual semiconducting and metallic nanowires. *Nat. Photon.* 2, 86-89 (2008).
6. P. J. Pauzauskie, A. Jamshidi, J. K. Valley, J. J. H. Satcher, M. C. Wu, Parallel trapping of multiwalled carbon nanotubes with optoelectronic tweezers. *Appl. Phys. Lett.* 95, 113104 (2009).
7. B. H. Lapizco-Encinas, On the recent developments of insulator-based dielectrophoresis: A review. *Electrophoresis* 40, 358-375 (2019).
8. C. Witte, J. Reboud, J. M. Cooper, S. L Neale, Channel integrated optoelectronic tweezer chip for microfluidic particle manipulation. *J. Micromech. Microeng.* 30, 045004 (2020).
9. J. K. Valley, A. Jamshidi, A. T. Ohta, H. Y. Hsu, M. C. Wu, Operational regimes and physics present in optoelectronic tweezers. *J Microelectromech. Syst.* 17, 342-350 (2008).
10. J. Happel, H. Brenner, The Behavior of Fluids in Slow Motion, *Low Reynolds number hydrodynamics.* Springer, Dordrecht, 23-57 (1983).
11. S. Zhang, E. Y. Scott, J. Singh, Y. Chen, Y. Zhang, M. Elsayed, M. D. Chamberlain, N. Shakiba, K. Adams, S. Yu, C. M. Morshead, P. W. Zandstra, A. R. Wheeler, The optoelectronic microrobot: A versatile toolbox for micromanipulation. *Proc. Natl. Acad. Sci. U.S.A.* 116, 14823-14828 (2019).
