# Supplementary material for: Reconfigurable multi-component micromachines driven by optoelectronic tweezers
Source: Nat Commun. 2021 Sep 9;12:5349. doi: 10.1038/s41467-021-25582-8 (PMC8429428; doi:10.1038/s41467-021-25582-8)
Supplement: Supplementary file 2 — Description of Additional Supplementary Files [file 41467_2021_25582_MOESM2_ESM.docx]

**Title:** Supplementary Movie 1

**Description:** A micro-gear is rotating at 360 deg/s (clip 1), 150 deg/s (clip 2) and 50 deg/s (clip 3).

**Title:** Supplementary Movie 2

**Description:** A micro-gear is translating at 800 μm/s (clip 1); a micro-gear is rotating (150 deg/s) and translating (300 μm/s) simultaneously (clip 2).

**Title:** Supplementary Movie 3

**Description:** Nine micro-gears are positioned into an array and made to rotate at different directions (clockwise or counter clockwise) and different angular velocities. At various stages, the light pattern is projected prior to applying the voltage. The micro-gears and particles only respond when both are present.

**Title:** Supplementary Movie 4

**Description:** A micro-gear fails in 'flipping' mode when rotated by an optical ring-spanner with no ring (clip 1) and with a thin ring (clip 2). A micro-gear fails in 'stripping' mode when rotated by an optical ring-spanner with thick ring (clip 3).

**Title:** Supplementary Movie 5

**Description:** Movement of suspensions of 1 μm- (clip 1) and 15 μm- (clip 2) diameter microbeads around a micro-gear rotating at 360 deg/s.

**Title:** Supplementary Movie 6

**Description:** A 7 μm microbead is made to revolve around a micro-gear at (i) 20 μm/s when the micro-gear rotates at 360 deg/s (clip 1), (ii) 7 μm/s when the micro-gear is rotates at 200 deg/s (clip 2), and (iii) at 0 μm/s (i.e., no revolution) when only the optical ring-spanner rotates (with no micro-gear) at 360 deg/s (clip 3).

**Title:** Supplementary Movie 7

**Description:** Time-dependent 3D simulated flow velocity distribution around a micro-gear rotating at 360 deg/s (with duration, acceleration, and saturation parameters given above), shown as a heat map from low (blue) to high (red).

**Title:** Supplementary Movie 8

**Description:** Time-dependent 2D-simulated flow velocity distribution around a micro-gear rotating at 360 deg/s (with duration, acceleration, and saturation parameters given above), shown as a heat map from low (blue) to high (red). This animation is an XY-slice of Aimation S1 at Z = 11 μm.

**Title:** Supplementary Movie 9

**Description:** A 20 μm-dia. microbead is propelled and accelerated by a touchless micro-feed-roller formed from a pair of micromotors rotating at 360 deg/s.

**Title:** Supplementary Movie 10

**Description:** A B16 cell is propelled by an OET-bridged touchless micro-feed-roller formed from a pair of micromotors rotating at 300 deg/s and translating at 35 μm/s (clip 1). A B16 cell is propelled by the light pattern (only), translating at 13 μm/s (clip 2).

**Title:** Supplementary Movie 11

**Description:** A doughnut-shaped OET trap is used to translate 15 μm dia. beads to a micro-wall and to a micro-plateau. In both cases, the beads are dislodged from the OET trap (clip 1). A stationary OET-bridged touchless micro-feed-roller (formed from a pair of micromotors rotating at 360 deg/s) causes 15 μm dia. beads to move foward and hop over a micro-wall, onto a micro-plateau, into a square micro-corral, and into a circular micro-corral (2 times). In each case, the light pattern is projected prior to applying the voltage, and the micro-gears and particles only respond when both are present (clip 2). The light pattern (only) causes a 15 μm dia. bead to hop up but not into a circular micro-corral (clip 3).

**Title:** Supplementary Movie 12

**Description:** Micro-gear-trains comprising one active micro-gear rotated at 100 deg/s driving one passive micro-gear (clip 1), and one active micro-gear rotated at 30 deg/s driving three passive micro-gears (clip 2).

**Title:** Supplementary Movie 13

**Description:** Micro-gear-trains comprising two active micro-gears rotated at 165 deg/s driving one passive micro-gear (clip 1), and four active micro-gears rotated at 100 deg/s driving three passive micro-gears (clip 2).

**Title:** Supplementary Movie 14

**Description:** Micro-gear-trains comprising one small active micro-gear rotated at 165 deg/s driving one large passive micro-gear (clip 1), one small active micro-gear rotated at 28 deg/s driving two large passive micro-gears (clip 2), and one large active micro-gear rotated at 100 deg/s driving one small passive micro-gear (clip 3)

**Title:** Supplementary Movie 15

**Description:** Micro-rack-and-pinion (alone) in which the pinion is rotated counter-clockwise (clip 1) or clockwise (clip 2). Micro-rack-and-pinion interfaced with the right side of a microchannel junction in which the active pinion is rotated counter-clockwise to push the rack into the junction (clip 3) or clockwise to pull the rack out of the junction (clip 4). Micro-rack-and-pinion interfaced with the left side of a microchannel junction in which the pinion is rotated clockwise to push the rack into the junction (clip 5) or counter-clockwise to pull the rack out of the junction (clip 6). Two micro-rack-and-pinion systems interfaced with a stationary microchannel junction in which the pinions are rotated (in opposite directions) to push the racks into the junction, closing the valve (clip 7), or to pull the racks out of the junction, opening the valve (clip 8).

**Title:** Supplementary Movie 16

**Description:** Suspension of 10 μm dia. polystyrene microbeads pumped through the microchannel junction (right to left) at 5 μL/min with no (micro-rack-and-pinion) valves controlling the flow (clip 1), valves positioned to isolate the flow to channel 2 (clip 2), and valves positioned to close all of the channels (clip 3).
